# Emergency Department Visits Involving Mental Health Conditions, Suicide-Related Behaviors, and Drug Overdoses Among Adolescents — United States, January 2019–February 2023

**DOI:** 10.15585/mmwr.mm7219a1

**Published:** 2023-05-12

**Authors:** Kayla N. Anderson, Dylan Johns, Kristin M. Holland, Yushiuan Chen, Alana M. Vivolo-Kantor, Eva Trinh, Rebecca H. Bitsko, Rebecca T. Leeb, Lakshmi Radhakrishnan, Sarah Bacon, Christopher M. Jones

**Affiliations:** ^1^National Center for Injury Prevention and Control, CDC; ^2^Office of Public Health Data, Surveillance, and Technology, CDC; ^3^ICF International, Atlanta, Georgia; ^4^National Center on Birth Defects and Developmental Disabilities, CDC.

The U.S. adolescent mental and behavioral health crisis is ongoing,* with high pre–COVID-19 pandemic baseline rates^†^ (*1*) and further increases in poor mental health (*2*), suicide-related behaviors (*3*), and drug overdose deaths (*4*) reported during 2020–2021. CDC examined changes in U.S. emergency department (ED) visits for mental health conditions (MHCs) overall and for nine specific MHCs,^§^ suicide-related behaviors (including suspected suicide attempts), and drug-involved overdoses (including opioids) among children and adolescents aged 12–17 years (adolescents) during January 2019–February 2023, overall and by sex. Compared with fall 2021, by fall 2022, decreases in weekly ED visits were reported among all adolescents, and females specifically, for MHCs overall, suicide-related behaviors, and drug overdoses; weekly ED visits among males were stable. During this same period, increases in weekly ED visits for opioid-involved overdoses were detected. Mean weekly ED visits in fall 2022 for suicide-related behaviors and MHCs overall were at or lower than the 2019 prepandemic baseline, respectively, and drug overdose visits were higher. Differences by sex were observed; levels among females were at or higher than prepandemic baselines for these conditions. These findings suggest some improvements as of fall 2022 in the trajectory of adolescent mental and behavioral health, as measured by ED visits; however, poor mental and behavioral health remains a substantial public health problem, particularly among adolescent females. Early identification and trauma-informed interventions, coupled with expanded evidence-based, comprehensive prevention efforts, are needed to support adolescents’ mental and behavioral health.

CDC examined ED visit data for adolescents from facilities consistently reporting data to the National Syndromic Surveillance Program (NSSP) during January 2019–early February 2023. A collaboration among CDC, local, and state health departments, and federal, academic, and private sector partners, NSSP receives anonymized medical record data from approximately 75% of EDs nationwide, although fewer than 50% of facilities from California, Hawaii, Minnesota, and Oklahoma currently participate. To reduce artifactual impact from changes in reporting patterns, analyses were restricted to facilities with a coefficient of variation for ED visits of ≤40 and average weekly informative discharge diagnosis ≥75% complete throughout the study period. In addition to displaying continuous trends, school semester surveillance periods in 2022 (spring included calendar weeks 1–23; summer, weeks 24–36; and fall, weeks 37–53) were compared with corresponding periods in 2021 and 2019 to monitor recent changes in ED visits and differences from the prepandemic baseline, respectively. School semester surveillance periods were used after visual inspection of visits related to MHCs, suicide-related behaviors, and drug overdoses for adolescents, which indicated substantial seasonal variation in visit patterns that mirrored U.S. K–12 education semesters (spring semester, summer vacation, fall semester). ED visits of interest were identified using a combination of free-text reason-for-visit (chief complaint), and administrative diagnosis codes (determined using codes from the *International Classification of Diseases, Ninth Edition, Clinical Modification*; *International Classification of Diseases, Tenth Edition, Clinical Modification*; and the Systematized Nomenclature of Medicine) (Supplementary Table, https://stacks.cdc.gov./view/cdc/127852), and did not differentiate by the primary or secondary diagnosis when multiple medical conditions were present as part of the visit record. CDC calculated percent change in mean weekly ED visits overall and by sex.^¶^ Changes were classified as decreased (≤−10%), stable (>−10% to <10%) or increased (≥10%) to support meaningful change identification and reduce identification of changes resulting from normative national ED visit fluctuations. Visit ratios (VRs)** with 95% CIs were calculated to describe the proportion of ED visits of interest among all adolescent ED visits in the surveillance versus comparison periods. Analyses were conducted using R software (version 4.1.2; The R Foundation). This activity was reviewed by CDC and conducted consistent with applicable federal law and policy.^††^

During January 2019–February 2023, adolescent ED visits for MHCs (overall and specific), suicide-related behaviors (including suspected suicide attempts), and drug overdoses (including opioid-involved overdoses) varied over time and by school semester (Figure) (Supplementary Figure, https://stacks.cdc.gov./view/cdc/127853). Mean weekly ED visits for MHCs overall, suicide-related behaviors, and drug overdoses were stable during spring and summer 2022 compared with those during 2021 (Table 1). By fall 2022, mean weekly adolescent ED visits were decreasing for MHCs overall (−11%), suicide-related behaviors (−12%), and drug overdoses (−10%) compared with fall 2021; trends for females mirrored overall patterns, whereas visits among males were stable for each of these outcomes (−7% to 3%). With some exceptions,^§§^ visits for MHCs overall, suicide-related behaviors, and all drug overdoses accounted for a smaller proportion of ED visits during 2022 compared with 2021.

**FIGURE:**
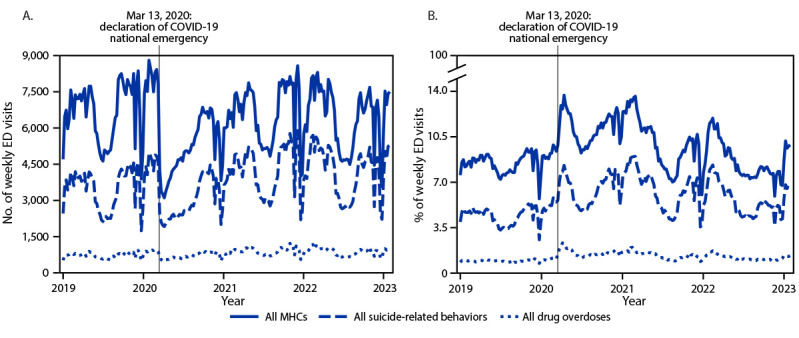
Mean weekly number (A) and percentage (B) of emergency department visits*^,^^†^ for mental health conditions overall,^§^ all suicide-related behaviors,^¶^ and all drug overdoses** among persons aged 12–17 years — National Syndromic Surveillance Program, United States, January 2019–February 2023^††^ **Abbreviations**: ED = emergency department; ICD-9-CM = *International Classification of Diseases, Ninth Edition, Clinical Modification*; ICD-10-CM = *International Classification of Diseases, Tenth Edition, Clinical Modification*; MHC = mental health condition; NSSP = National Syndromic Surveillance Program; SNOMED = Systematized Nomenclature of Medicine. * NSSP receives anonymized medical record information from approximately 75% of nonfederal EDs nationwide. NSSP collects free-text reason-for-visit (chief complaint), discharge diagnosis, and patient demographic details. Diagnosis information is collected using ICD-9-CM, ICD-10-CM, and SNOMED codes. ^†^ To reduce artifactual impact from changes in reporting patterns, analyses were restricted to facilities with a coefficient of variation for ED visits ≤40 and average weekly informative discharge diagnosis ≥75% complete throughout the study period. ^§^ The overall MHC classification identifies any mental health-related ED visits, including those for the nine MHCs included in this analysis (anxiety, attention-deficit/hyperactivity disorders, bipolar disorders, depression, disruptive behavioral and impulse-control disorders, eating disorders, obsessive-compulsive disorders, tic disorders, and trauma and stressor-related disorders), schizophrenia spectrum disorders, additional low-prevalence MHCs (e.g., delusional disorders and reactive attachment), and general mental health terms and codes. ^¶^ The suicide-related behaviors classification identifies ED visits related to suicidal ideation, self-harm, and suspected suicide attempts. ** The drug overdose classification identifies acute drug poisonings from any type of drug. ^††^ The time series displays data from epidemiologic week 1 for 2019 (December 30, 2018) through epidemiologic week 5 for 2023 (February 4, 2023).

**TABLE 1:** Changes in mean weekly number and percentage of emergency department visits*^,^^†^ involving overall^§^ and specific mental health conditions, suicide-related behaviors including suspected suicide attempts,^¶^ and all drug overdoses including opioid-involved overdoses** among persons aged 12–17 years, by school semester — National Syndromic Surveillance Program, United States, 2021–2022^††^^,^^§§^

Mental and behavioral health indicator/Sex	Surveillance period Comparison period								
	Spring semester, 2022 (weeks 1–23)^††^ Spring semester, 2021 (weeks 1–23)			Summer, 2022 (weeks 24–36)^††^ Summer, 2021 (weeks 24–36)			Fall semester, 2022 (weeks 37–53)^††^ Fall semester, 2021 (weeks 37–53)		
	Mean weekly ED visit counts, surveillance period	Absolute change in mean weekly ED visit counts^¶¶^ (%)	VR (95% CI)***	Mean weekly ED visit counts, surveillance period	Absolute change in mean weekly ED visit counts^¶¶^ (%)	VR (95% CI)***	Mean weekly ED visit counts, surveillance period	Absolute change in mean weekly ED visit counts^¶¶^ (%)	VR (95% CI)***
**Overall mental health conditions**									
**All**	**7,083**	**273 (4)**	**0.83 (0.83–0.84)**	**5,031**	**−385 (−7)**	**0.97 (0.96–0.98)**	**6,441**	**−767 (−11)**	**0.82 (0.81–0.82)**
Female	4,572	70 (2)	0.83 (0.82–0.84)	3,166	−337 (−10)	0.96 (0.95–0.97)	4,057	−616 (−13)	0.81 (0.80–0.82)
Male	2,493	200 (9)	0.85 (0.84–0.86)	1,850	−51 (−3)	0.99 (0.97–1.01)	2,366	−155 (−6)	0.84 (0.82–0.85)
**Anxiety disorders**									
**All**	**2,104**	**−4 (—)**	**0.80 (0.79–0.81)**	**1,697**	**−140 (−8)**	**0.96 (0.95–0.98)**	**1,874**	**−263 (−12)**	**0.80 (0.79–0.81)**
Female	1,486	−16 (−1)	0.81 (0.80–0.82)	1,181	−122 (−9)	0.96 (0.94–0.99)	1,303	−206 (−14)	0.81 (0.79–0.82)
Male	609	11 (2)	0.80 (0.78–0.82)	508	−20 (−4)	0.98 (0.95–1.01)	562	−60 (−10)	0.80 (0.78–0.83)
**Depressive disorders**									
**All**	**3,055**	**−66 (−2)**	**0.78 (0.78–0.79)**	**1,801**	**−330 (−15)**	**0.88 (0.87–0.90)**	**2,584**	**−581 (−18)**	**0.74 (0.74–0.75)**
Female	2,202	−102 (−4)	0.78 (0.77–0.79)	1,284	−287 (−18)	0.87 (0.85–0.89)	1,824	−475 (−21)	0.74 (0.73–0.75)
Male	844	35 (4)	0.82 (0.80–0.83)	511	−43 (−8)	0.94 (0.91–0.97)	751	−106 (−12)	0.78 (0.76–0.80)
**Attention-deficit/Hyperactivity disorders**									
**All**	**794**	**−16 (−2)**	**0.79** **(0.77–0.80**	**622**	**−52 (−8)**	**0.96 (0.93–0.99)**	**737**	**−97 (−12)**	**0.81 (0.79–0.83)**
Female	318	−18 (−5)	0.77 (0.75–0.80)	245	−22 (−8)	0.98 (0.93–1.02)	291	−47 (−14)	0.80 (0.77–0.83)
Male	472	1 (—)	0.79 (0.76–0.81)	372	−32 (−8)	0.94 (0.90–0.97)	442	−51 (−10)	0.80 (0.77–0.82)
**Trauma and stressor-related disorders**									
**All**	**803**	**82 (11)**	**0.89 (0.87–0.91)**	**562**	**−17 (−3)**	**1.01 (0.98–1.05)**	**744**	**−51 (−6)**	**0.85 (0.83–0.88)**
Female	533	41 (8)	0.88 (0.86–0.91)	371	−21 (−5)	1.01 (0.97–1.05)	481	−48 (−9)	0.85 (0.82–0.87)
Male	265	40 (18)	0.92 (0.89–0.96)	187	3 (2)	1.04 (0.98–1.10)	260	−2 (−1)	0.88 (0.85–0.92)
**Disruptive behavioral and impulse disorders**									
**All**	**514**	**45 (10)**	**0.88 (0.86–0.90)**	**391**	**−18 (−4)**	**1.00 (0.96–1.04)**	**458**	**−50 (−10)**	**0.82 (0.80–0.85)**
Female	230	17 (8)	0.88 (0.85–0.92)	178	−4 (−2)	1.04 (0.98–1.10)	209	−18 (−8)	0.86 (0.82–0.90)
Male	282	28 (11)	0.87 (0.84–0.90)	211	−14 (−6)	0.95 (0.90–1.00)	247	−33 (−12)	0.79 (0.75–0.82)
**Bipolar disorders**									
**All**	**229**	**−23 (−9)**	**0.73 (0.70–0.75)**	**183**	**−29 (−14)**	**0.90 (0.85–0.95)**	**201**	**−32 (−14)**	**0.79 (0.75–0.82)**
Female	148	−12 (−7)	0.76 (0.72–0.79)	114	−20 (−15)	0.91 (0.84–0.97)	130	−20 (−13)	0.81 (0.76–0.86)
Male	80	−12 (−13)	0.68 (0.64–0.72)	68	−10 (−12)	0.89 (0.82–0.98)	70	−12 (−15)	0.75 (0.70–0.82)
**Eating disorders**									
**All**	**141**	**4 (3)**	**0.83 (0.79–0.87)**	**104**	**−21 (−17)**	**0.86 (0.80–0.93)**	**115**	**−29 (−20)**	**0.73 (0.69–0.77)**
Female	124	0 (—)	0.82 (0.78–0.86)	91	−20 (−18)	0.87 (0.81–0.94)	100	−28 (−22)	0.73 (0.68–0.78)
Male	15	4 (34)	1.05 (0.89–1.23)	12	−1 (−9)	0.92 (0.74–1.15)	14	−1 (−6)	0.84 (0.70–1.01)
**Tic disorders**									
**All**	**42**	**−24 (−36)**	**0.51 (0.47–0.55)**	**30**	**−10 (−25)**	**0.78 (0.69–0.89)**	**34**	**−19 (−36)**	**0.59 (0.53–0.65)**
Female	27	−23 (−46)	0.44 (0.40–0.49)	19	−9 (−32)	0.72 (0.61–0.84)	19	−16 (−46)	0.50 (0.44–0.58)
Male	15	−1 (−9)	0.72 (0.62–0.83)	11	−1 (−7)	0.95 (0.75–1.20)	14	−2 (−14)	0.77 (0.64–0.91)
**Obsessive-compulsive disorders**									
**All**	**49**	**−5 (−10)**	**0.72 (0.67–0.78)**	**41**	**−5 (−11)**	**0.93 (0.83–1.04)**	**43**	**−8 (−16)**	**0.77 (0.69–0.85)**
Female	29	−2 (−8)	0.75 (0.68–0.84)	24	0 (—)	1.06 (0.91–1.24)	26	−3 (−9)	0.85 (0.74–0.97)
Male	20	−3 (−11)	0.70 (0.61–0.79)	17	−5 (−23)	0.78 (0.66–0.94)	17	−6 (−26)	0.66 (0.57–0.78)
**Suicide-related behaviors**									
**All**	**4,699**	**328 (8)**	**0.86 (0.85–0.87)**	**2,967**	**−196 (−6)**	**0.98 (0.96–0.99)**	**4,219**	**−570 (−12)**	**0.80 (0.80–0.81)**
Female	3,329	131 (4)	0.85 (0.84–0.86)	2,080	−203 (−9)	0.97 (0.95–0.98)	2,943	−478 (−14)	0.80 (0.79–0.81)
Male	1,360	195 (17)	0.92 (0.90–0.93)	880	6 (1)	1.03 (1.00–1.05)	1,267	−91 (−7)	0.83 (0.81–0.85)
**Suspected suicide attempts**									
**All**	**1,213**	**−36 (−3)**	**0.78 (0.77–0.79)**	**843**	**−96 (−10)**	**0.94 (0.91–0.96)**	**1,038**	**−220 (−17)**	**0.75 (0.74–0.77)**
Female	954	−58 (−6)	0.77 (0.76–0.78)	660	−95 (−13)	0.93 (0.90–0.96)	814	−185 (−19)	0.76 (0.74–0.78)
Male	256	23 (10)	0.86 (0.83–0.89)	181	−1 (−1)	1.01 (0.96–1.07)	222	−34 (−13)	0.77 (0.74–0.81)
**Drug overdoses overall**									
**All**	**961**	**40 (4)**	**0.84 (0.82–0.85)**	**704**	**−47 (−6)**	**0.98 (0.95–1.01)**	**862**	**−97 (−10)**	**0.82 (0.80–0.84)**
Female	690	4 (1)	0.82 (0.80–0.84)	496	−53 (−10)	0.96 (0.93–0.99)	604	−104 (−15)	0.80 (0.77–0.82)
Male	269	36 (15)	0.90 (0.87–0.94)	207	5 (3)	1.05 (0.99–1.10)	258	7 (3)	0.92 (0.88–0.96)
**Opioid-involved overdoses**									
**All**	**36**	**2 (7)**	**0.86 (0.78–0.95)**	**38**	**4 (12)**	**1.17 (1.03–1.33)**	**40**	**8 (27)**	**1.16 (1.03–1.30)**
Female	17	2 (17)	0.96 (0.83–1.11)	16	1 (6)	1.12 (0.92–1.37)	16	1 (10)	1.03 (0.86–1.22)
Male	19	0 (−1)	0.78 (0.68–0.88)	22	3 (16)	1.18 (1.00–1.40)	23	7 (41)	1.25 (1.07–1.47)

**Abbreviations:** ED = emergency department; ICD-9-CM = *International Classification of Diseases, Ninth Edition, Clinical Modification*; ICD-10-CM = *International Classification of Diseases, Tenth Edition, Clinical Modification*; MHC = mental health condition; NSSP = National Syndromic Surveillance Program; SNOMED = Systematized Nomenclature of Medicine.

* NSSP receives anonymized medical record information from approximately 75% of nonfederal EDs nationwide. NSSP collects free-text reason-for-visit (chief complaint), discharge diagnosis, and patient demographic details. Diagnosis information is collected using ICD-9-CM, ICD-10-CM, and SNOMED codes.

^†^ To reduce artifactual impact from changes in reporting patterns, analyses were restricted to facilities with a coefficient of variation for ED visits ≤40 and average weekly informative discharge diagnosis ≥75% complete throughout the study period.

^§^ The overall MHC classification identifies all mental health–related ED visits, including the nine MHCs included in this analysis (anxiety, attention-deficit/hyperactivity disorders, bipolar disorders, depression, disruptive behavioral and impulse-control disorders, eating disorders, obsessive-compulsive disorders, tic disorders, and trauma and stressor-related disorders), schizophrenia spectrum disorders, additional low-prevalence MHCs (e.g., delusional disorders and reactive attachment), and general mental health terms and codes.

^¶^ The suicide-related behaviors classification identifies ED visits related to suicidal ideation, self-harm, and suspected suicide attempts, whereas the suspected suicide attempt classification only includes suspected suicide attempts.

** The drug overdose classification identifies acute drug poisonings from any type of drug, whereas the opioid-involved overdose classification includes acute drug poisonings from illicit (e.g., heroin) or prescription opioids (e.g., oxycodone).

^††^ School semester surveillance periods during 2022 were as follows: spring, calendar weeks 1–23 (Jan 2–Jun 11, 2022); summer, calendar weeks 24–36 (Jun 12–Sep 10, 2022); and fall, calendar weeks 37–53 (Sep 11–Dec 31, 2022). Corresponding school semester comparison periods during 2021 were as follows: spring, calendar weeks 1–23 (Jan 3–Jun 12, 2021); summer, calendar weeks 24–36 (Jun 13–Sep 11, 2021); and fall, calendar weeks 37–53 (Sep 12, 2021–Jan 1, 2022).

^§§^ Individual values for females and males might not add up to the total values because of rounding.

^¶¶^ Percent change in visits per week during each surveillance period was calculated as the difference in mean weekly visits between the surveillance period and the comparison period, divided by the mean weekly visits during the comparison period, x 100% ([{mean weekly ED visits with condition of interest during surveillance period − mean weekly ED visits with condition of interest during comparison period} / mean weekly ED visits with condition of interest during the comparison period] x 100%).

*** VR is the proportion of ED visits with condition of interest during the surveillance period, divided by the proportion of ED visits with condition of interest during the comparison period ([ED visits with condition of interest {surveillance period} / all ED visits {surveillance period}] / [ED visits with condition of interest {comparison period} / all ED visits {comparison period}]). Ratios >1 indicate a higher proportion of ED visits with the condition of interest during the surveillance period compared with the comparison period; ratios <1 indicate a lower proportion during the surveillance period compared with the comparison period.

From school semesters in 2021 to those in 2022, variation in ED visits for specific MHCs, suspected suicide attempts, and opioid-involved overdoses overall and by sex were observed (Table 1). By fall 2022, compared with fall 2021, mean weekly ED visits for opioid-involved overdoses increased among both females (10%) and males (41%). Compared with the same periods, ED visits for specific MHCs and suspected suicide attempts among females generally mirrored trends in visits for overall MHCs and suicide-related behaviors. Among males, mean weekly ED visits were stable for MHCs overall (−6%) and suicide-related behaviors (−7%), but decreased for some specific MHCs (e.g., anxiety [−10%], depression [−12%], and many less common conditions) and suspected suicide attempts (−13%). Among all adolescent ED visits, those for specific MHCs and suspected suicide attempts accounted for a smaller proportion (VRs = 0.59–0.85 and 0.75, respectively), and opioid-involved overdoses for a larger proportion (VR = 1.16) during fall 2022 compared with fall 2021. With some exceptions, sex-stratified findings were generally similar to these overall trends.

Compared with those during 2019 school semesters, visits for MHCs overall, suicide-related behaviors, and drug overdoses during 2022 varied (Table 2). By fall 2022, compared with fall 2019, mean weekly ED visits were lower than the prepandemic baseline for MHCs overall (–13%) and comparable to baseline for suicide-related behaviors (7%); visits for drug overdoses were higher during fall 2022 (10%) than during fall 2019. Mean weekly ED visits among females were stable for MHCs overall (−8%) but increased for suicide-related behaviors (14%) and drug overdoses (16%) during fall 2022 compared with fall 2019. Among males, mean weekly ED visits in fall 2022 for MHCs overall were lower (−20%) than those during fall 2019, but were stable for suicide-related behaviors (−6%) and drug overdoses (−3%). Among all adolescent ED visits during fall 2022, those for MHCs overall accounted for a lower proportion (VR = 0.87), and those for suicide-related behaviors and drug overdoses for a higher proportion (VRs = 1.07 and 1.10, respectively) than during fall 2019. In fall 2022, VR findings by sex generally mirrored broader trends, especially for females; among males, the proportion of suicide-related behaviors was lower (VR = 0.94) and for drug overdose (VR = 0.97) was similar, compared with fall 2019.

**TABLE 2:** Mean weekly number and percentage of emergency department visits*^,^^†^ involving overall^§^ and specific mental health conditions, suicide-related behaviors including suspected suicide attempts,^¶^ and all drug overdoses including opioid-involved overdoses** among persons aged 12–17 years — National Syndromic Surveillance Program, United States, 2019^††^ and 2022^§§^

Mental and behavioral health indicator/Sex	Surveillance period Comparison period								
	Spring semester, 2022 (weeks 1–23)^††^ Spring semester, 2019 (weeks 1–23)			Summer, 2022 (weeks 24–36)^††^ Summer, 2019 (weeks 24–36)			Fall semester, 2022 (weeks 37–53)^††^ Fall semester, 2019 (weeks 37–53)		
	Mean weekly ED visit counts, surveillance period	Absolute change in mean weekly ED visit counts^¶¶^ (%)	VR (95% CI)***	Mean weekly ED visit counts, surveillance period	Absolute change in mean weekly ED visit counts^¶¶^ (%)	VR (95% CI)***	Mean weekly ED visit counts, surveillance period	Absolute change in mean weekly ED visit count)^¶¶^ (%)	VR (95% CI)***
**Overall mental health conditions**									
**All**	**7,083**	**252 (4)**	**1.13 (1.13–1.14)**	**5,031**	**−279 (−5)**	**0.97 (0.96–0.98)**	**6,441**	**−943 (−13)**	**0.87 (0.86–0.88)**
Female	4,572	462 (11)	1.22 (1.21–1.23)	3,166	8 (—)	1.04 (1.03–1.05)	4,057	−343 (−8)	0.93 (0.92–0.94)
Male	2,493	−213 (−8)	1.01 (1.00–1.02)	1,850	−294 (−14)	0.88 (0.87–0.89)	2,366	−606 (−20)	0.79 (0.78–0.80)
**Anxiety disorders**									
**All**	**2,104**	**159 (8)**	**1.18 (1.17–1.20)**	**1,697**	**−94 (−5)**	**0.98 (0.96–0.99)**	**1,874**	**−331 (−15)**	**0.85 (0.84–0.86)**
Female	1,486	164 (12)	1.23 (1.21–1.25)	1,181	−28 (−2)	1.01 (0.99–1.04)	1,303	−192 (−13)	0.88 (0.86–0.89)
Male	609	−8 (−1)	1.08 (1.06–1.11)	508	−69 (−12)	0.90 (0.87–0.93)	562	−145 (−20)	0.79 (0.77–0.81)
**Depressive disorders**									
**All**	**3,055**	**156 (5)**	**1.15 (1.14–1.16)**	**1,801**	**−116 (−6)**	**0.97 (0.95–0.98)**	**2,584**	**−528 (−17)**	**0.83 (0.82–0.84)**
Female	2,202	232 (12)	1.22 (1.21–1.24)	1,284	−32 (−2)	1.01 (0.99–1.03)	1,824	−278 (−13)	0.87 (0.86–0.89)
Male	844	−80 (−9)	1.00 (0.98–1.02)	511	−86 (−14)	0.87 (0.85–0.90)	751	−251 (−25)	0.74 (0.73–0.76)
**Attention-deficit/Hyperactivity disorders**									
**All**	**794**	**−236 (−23)**	**0.84 (0.83–0.86)**	**622**	**−290 (−32)**	**0.70 (0.68–0.72)**	**737**	**−445 (−38)**	**0.62 (0.61–0.64)**
Female	318	−55 (−15)	0.93 (0.90–0.96)	245	−92 (−27)	0.75 (0.72–0.79)	291	−139 (−32)	0.68 (0.66–0.71)
Male	472	−181 (−28)	0.79 (0.77–0.81)	372	−200 (−35)	0.66 (0.64–0.69)	442	−308 (−41)	0.58 (0.57–0.60)
**Trauma and stressor-related disorders**									
**All**	**803**	**69 (9)**	**1.20 (1.17–1.22)**	**562**	**−2 (—)**	**1.03 (0.99–1.06)**	**744**	**−64 (−8)**	**0.92 (0.90–0.94)**
Female	533	69 (15)	1.26 (1.22–1.29)	371	6 (2)	1.06 (1.01–1.10)	481	−26 (−5)	0.95 (0.93–0.98)
Male	265	−3 (−1)	1.08 (1.05–1.12)	187	−10 (−5)	0.97 (0.92–1.02)	260	−40 (−13)	0.86 (0.82–0.90)
**Disruptive behavioral and impulse disorders**									
**All**	**514**	**−66 (−11)**	**0.97 (0.95–0.99)**	**391**	**−102 (−21)**	**0.82 (0.79–0.85)**	**458**	**−148 (−24)**	**0.75 (0.73–0.78)**
Female	230	−16 (−6)	1.02 (0.99–1.06)	178	−33 (−16)	0.87 (0.83–0.92)	209	−46 (−18)	0.82 (0.79–0.86)
Male	282	−49 (−15)	0.93 (0.90–0.96)	211	−69 (−25)	0.77 (0.73–0.81)	247	−102 (−29)	0.70 (0.67–0.73)
**Bipolar disorders**									
**All**	**229**	**−61 (−21)**	**0.86 (0.83–0.90)**	**183**	**−86 (−32)**	**0.70 (0.67–0.74)**	**201**	**−116 (−37)**	**0.63 (0.61–0.66)**
Female	148	−26 (−15)	0.93 (0.89–0.98)	114	−50 (−30)	0.72 (0.68–0.77)	130	−61 (−32)	0.69 (0.65–0.72)
Male	80	−35 (−31)	0.76 (0.71–0.80)	68	−37 (−35)	0.66 (0.61–0.72)	70	−55 (−44)	0.55 (0.52–0.60)
**Eating disorders**									
**All**	**141**	**75 (114)**	**2.34 (2.20–2.49)**	**104**	**44 (72)**	**1.77 (1.62–1.93)**	**115**	**41 (55)**	**1.55 (1.44–1.66)**
Female	124	68 (121)	2.42 (2.27–2.58)	91	39 (75)	1.82 (1.65–2.00)	100	36 (57)	1.57 (1.46–1.70)
Male	15	6 (61)	1.76 (1.49–2.09)	12	4 (46)	1.49 (1.16–1.90)	14	4 (37)	1.36 (1.11–1.67)
**Tic disorders**									
**All**	**42**	**13 (44)**	**1.57 (1.43–1.74)**	**30**	**5 (20)**	**1.24 (1.07–1.43)**	**34**	**2 (8)**	**1.07 (0.95–1.21)**
Female	27	14 (108)	2.27 (1.98–2.61)	19	9 (93)	2.00 (1.62–2.47)	19	7 (56)	1.57 (1.31–1.88)
Male	15	−2 (−9)	0.99 (0.85–1.15)	11	−4 (−29)	0.73 (0.59–0.90)	14	−5 (−24)	0.75 (0.63–0.89)
**Obsessive-compulsive disorders**									
**All**	**49**	**1 (2)**	**1.12 (1.03–1.22)**	**41**	**−1 (−3)**	**1.00 (0.89–1.13)**	**43**	**−10 (−19)**	**0.81 (0.73–0.90)**
Female	29	6 (28)	1.40 (1.25–1.58)	24	3 (16)	1.20 (1.02–1.41)	26	0 (−2)	0.99 (0.86–1.13)
Male	20	−5 (−21)	0.86 (0.76–0.98)	17	−4 (−21)	0.80 (0.67–0.96)	17	−10 (−36)	0.63 (0.54–0.74)
**Suicide-related behaviors**									
**All**	**4,699**	**1,008 (27)**	**1.39 (1.38–1.40)**	**2,967**	**505 (20)**	**1.24 (1.22–1.26)**	**4,219**	**292 (7)**	**1.07 (1.06–1.08)**
Female	3,329	867 (35)	1.48 (1.46–1.49)	2,080	446 (27)	1.32 (1.30–1.34)	2,943	363 (14)	1.15 (1.13–1.16)
Male	1,360	137 (11)	1.22 (1.20–1.24)	880	56 (7)	1.09 (1.06–1.12)	1,267	−74 (−6)	0.94 (0.92–0.95)
**Suspected suicide attempts**									
**All**	**1,213**	**328 (37)**	**1.50 (1.47–1.53)**	**843**	**165 (24)**	**1.28 (1.24–1.32)**	**1,038**	**121 (13)**	**1.13 (1.11–1.15)**
Female	954	285 (43)	1.56 (1.53–1.59)	660	150 (30)	1.34 (1.30–1.39)	814	131 (19)	1.20 (1.17–1.23)
Male	256	41 (19)	1.30 (1.25–1.35)	181	14 (8)	1.10 (1.04–1.17)	222	−12 (−5)	0.94 (0.90–0.98)
**Drug overdoses overall**									
**All**	**961**	**208 (28)**	**1.40 (1.37–1.42)**	**704**	**96 (16)**	**1.19 (1.16–1.23)**	**862**	**78 (10)**	**1.10 (1.07–1.12)**
Female	690	180 (35)	1.48 (1.45–1.52)	496	87 (21)	1.26 (1.21–1.30)	604	84 (16)	1.17 (1.13–1.20)
Male	269	27 (11)	1.22 (1.17–1.26)	207	8 (4)	1.06 (1.01–1.12)	258	−7 (−3)	0.97 (0.92–1.01)
**Opioid-involved overdoses**									
**All**	**36**	**15 (73)**	**1.89 (1.69–2.12)**	**38**	**21 (123)**	**2.30 (1.96–2.69)**	**40**	**12 (44)**	**1.44 (1.28–1.63)**
Female	17	7 (70)	1.86 (1.58–2.20)	16	8 (103)	2.11 (1.66–2.67)	16	4 (32)	1.32 (1.10–1.59)
Male	19	8 (75)	1.91 (1.64–2.23)	22	13 (137)	2.42 (1.96–3.00)	23	8 (54)	1.53 (1.30–1.80)

**Abbreviations:** ED = emergency department; ICD-9-CM = *International Classification of Diseases, Ninth Edition, Clinical Modification*; ICD-10-CM = *International Classification of Diseases, Tenth Edition, Clinical Modification*; MHC = mental health condition; NSSP = National Syndromic Surveillance Program; SNOMED = Systematized Nomenclature of Medicine.

* NSSP receives anonymized medical record information from approximately 75% of nonfederal EDs nationwide. NSSP collects free-text reason-for-visit (chief complaint), discharge diagnosis, and patient demographic details. Diagnosis information is collected using ICD-9-CM, ICD-10-CM, and SNOMED codes.

^†^ To reduce artifactual impact from changes in reporting patterns, analyses were restricted to facilities with a coefficient of variation for ED visits ≤40 and average weekly informative discharge diagnosis ≥75% complete throughout the study period.

^§^ The overall MHC classification identifies all mental health–related ED visits, including the nine MHCs included in this analysis (anxiety, attention-deficit/hyperactivity disorders, bipolar disorders, depression, disruptive behavioral and impulse-control disorders, eating disorders, obsessive-compulsive disorders, tic disorders, and trauma and stressor-related disorders), schizophrenia spectrum disorders, additional low-prevalence MHCs (e.g., delusional disorders and reactive attachment), and general mental health terms and codes.

^¶^ The suicide-related behaviors classification identifies ED visits related to suicidal ideation, self-harm, and suspected suicide attempts, whereas the suspected suicide attempt classification only includes suspected suicide attempts.

** The drug overdose classification identifies acute drug poisonings from any type of drug, whereas the opioid-involved overdose classification includes acute drug poisonings from illicit (e.g., heroin) or prescription opioids (e.g., oxycodone).

^††^ School semester surveillance periods during 2022 were as follows: spring, calendar weeks 1–23 (Jan 2–Jun 11, 2022); summer, calendar weeks 24–36 (Jun 12–Sep 10, 2022); and fall, calendar weeks 37–53 (Sep 11–Dec 31, 2022). Corresponding school semester comparison periods during 2019 were as follows: spring, calendar weeks 1–23 (Dec 30, 2018–June 8, 2019); summer, calendar weeks 24–36 (June 9–Sept 7, 2019); and fall, calendar weeks 37–53 (Sept 8–Dec 28, 2019).

^§§^ Individual values for females and males might not add up to the total values because of rounding.

^¶¶^ Percent change in visits per week during each surveillance period was calculated as the difference in mean weekly visits between the surveillance period and the comparison period, divided by the mean weekly visits during the comparison period, x 100% ([{mean weekly ED visits with condition of interest during surveillance period − mean weekly ED visits with condition of interest during comparison period} / mean weekly ED visits with condition of interest during comparison period] x 100%).

*** VR is the proportion of ED visits with condition of interest during the surveillance period, divided by the proportion of ED visits with condition of interest during the comparison period ([ED visits with condition of interest {surveillance period} / all ED visits {surveillance period}] / (ED visits with condition of interest {comparison period} / all ED visits {comparison period}]). Ratios >1 indicate a higher proportion of ED visits with the condition of interest during the surveillance period compared with the comparison period; ratios <1 indicate a lower proportion during the surveillance period compared with the comparison period.

Adolescent ED visits for specific MHCs, suspected suicide attempts, and opioid-involved overdoses, overall and by sex, varied by school semester in 2022 compared with 2019 (Table 2). As of fall 2022, ED visits for eating disorders increased overall (55%; VR = 1.55) and for both sexes, and tic disorders increased among females only (56%; VR = 1.57). ED visits for other specific MHCs were lower than or comparable with visits during fall 2019. Patterns for suspected suicide attempts and opioid-involved overdoses generally followed the broader directional trends for suicide-related behaviors and drug overdoses, respectively.

## Discussion

These findings extend previous research that indicated worsening in some aspects of adolescent mental and behavioral health during the COVID-19 pandemic (*2*–*5*) and suggest some improvements in the trajectory of adolescent mental and behavioral health, as measured by ED visits. Declines in adolescent ED visits for overdoses overall from 2021 to 2022 are consistent with other available nonfatal^¶¶^ and provisional fatal overdose*** data, though comparable data beyond 2021 on mental health and suicidal behaviors are limited. Increases in opioid-involved overdoses warrant further investigation but might be related to the overall rarity of adolescent opioid-involved overdoses, such that even a 10% change actually represents a small absolute change in the number of overdoses. Still, any adolescent overdose is concerning, particularly as increased availability of highly potent and lethal counterfeit pills containing illicitly manufactured fentanyl among adolescents via social media platforms^†††^ has heighted awareness recently about increasing overdose risk among younger populations. Despite some recent declines in ED visits for MHCs, suicide-related behaviors, and drug overdoses, poor adolescent mental and behavioral health remains a notable public health problem (*1*–*6*), particularly because ED visits for these conditions remain similar to or higher than already concerningly high prepandemic baselines among females into 2022.

Multiple reasons might account for these findings. Many adolescents have returned to prepandemic-like school and community environments, which might have improved social engagement, reduced isolation, and supported mental and behavioral health for some adolescents (*6*,*7*). Familial or other stressors might also have declined, resulting in fewer adverse childhood experiences,^§§§^ which are strongly associated with adolescent mental and behavioral health (*8*). CDC has released resources to guide states, communities, and schools in selecting strategies for prevention of suicide,^¶¶¶^ overdose,**** and adverse childhood experiences,^††††^ based on the best available evidence. Implementation of these strategies and approaches, and others that support adolescents and their families^§§§§^ might improve mental and behavioral health for some adolescents. For example, communication campaigns^¶¶¶¶^ can improve the rapid identification of behavioral changes, improve adolescent help-seeking behaviors, and support early intervention by parents and trusted adults. Further, federal investments, such as the 988 suicide crisis line***** and improvements to accessible behavioral health care (e.g., telehealth)^†††††^ might have improved families’ ability to identify support before a crisis or get care outside EDs.

Clinicians who work with adolescents being treated in EDs for opioid overdose might consider screening for opioid use disorder and providing timely, FDA-approved medications (*9*); clinicians might also consider screening for depression and anxiety when evaluating adolescents.^§§§§§^ Continued promotion of policies and programs that improve access to mental and behavioral health services, coupled with primary prevention efforts that support adolescents and their families, might mitigate risk for mental and behavioral health problems before they begin (*10*). Further prevention, intervention, and response efforts can be implemented to continue improving adolescent mental and behavioral health.

The findings in this report are subject to at least five limitations. First, NSSP data are not nationally representative and data quality variations across facilities could potentially lead to over- or underreporting, potentially affecting visit trends. Second, this analysis used percent change thresholds to support identification of meaningful changes; however, this might under-identify (in the case of common ED visits such as overall MHCs) or over-identify (in the case of rare ED visits such as opioid-involved overdose) concerning trends, because this metric depends upon number of visits for conditions of interest. Third, these data cannot be used to make causal inferences regarding trend changes. Fourth, this analysis could not differentiate between primary or secondary diagnoses when multiple conditions were addressed at the visit. Finally, data are from ED visits which do not represent the full spectrum of adolescent mental and behavioral health challenges; trends warrant confirmation with adolescent self-report data.

Prioritizing implementation of evidence-based prevention and trauma-informed early intervention and treatment strategies that promote mental and behavioral health among adolescents might help prevent MHCs, suicide-related behaviors, and drug overdoses, and improve overall health. CDC supports efforts to promote adolescent well-being and provides resources for clinicians,^¶¶¶¶¶^ families,****** schools,^††††††^ and communities.^§§§§§§^

**Summary**

**What is already known about this topic?**

High baseline rates of poor adolescent mental and behavioral health were exacerbated by the COVID-19 pandemic.

**What is added by this report?**

By fall 2022, weekly ED visits among adolescents, and females in particular, for mental health conditions overall, suicide-related behaviors, and drug overdoses decreased compared with those during fall 2021; weekly ED visits among males were stable. Although sex differences were observed, as of fall 2022, weekly ED visits among females were at or higher than the prepandemic baseline for mental health conditions overall, suicide-related behaviors, and drug overdoses.

**What are the implications for public health practice?**

Early condition identification and trauma-informed interventions, coupled with evidence-based, comprehensive prevention efforts, are needed to support adolescents’ mental and behavioral health.

## Footnotes

* Multiple declarations related to the adolescents’ mental and behavioral health crisis have been issued by federal authorities (https://www.hhs.gov/sites/default/files/surgeon-general-youth-mental-health-advisory.pdf) and national health care organizations (https://www.aap.org/en/advocacy/child-and-adolescent-healthy-mental-development/aap-aacap-cha-declaration-of-a-national-emergency-in-child-and-adolescent-mental-health/).

† CDC data on adolescent mental and behavioral health can be queried online, including data on fatal and nonfatal overdoses and suicide-related behaviors (https://www.cdc.gov/injury/wisqars/index.html) and self-reported data on mental health, suicide-related behaviors, and substance use (https://nccd.cdc.gov/youthonline/App/Default.aspx).

§ Anxiety, attention-deficit/hyperactivity disorders, bipolar disorders, depression, disruptive behavioral and impulse-control disorders, eating disorders, obsessive-compulsive disorders, tic disorders, and trauma and stressor-related disorders.

¶ Percent change in weekly ED visits = ([mean weekly ED visits with condition of interest during surveillance period − mean weekly ED visits with condition of interest during comparison period] / mean weekly ED visits with condition of interest during comparison period) x 100%.

** VRs are the proportion of ED visits with condition of interest during the surveillance period, divided by the proportion of ED visits with condition of interest during the comparison period ([ED visits with condition of interest {surveillance period} / all ED visits {surveillance period}] / [ED visits with condition of interest {comparison period} / all ED visits {comparison period}]). Ratios >1 indicate a higher proportion of ED visits with the condition of interest during the surveillance period compared with the comparison period; ratios <1 indicate a lower proportion during the surveillance period compared with the comparison period.

†† 45 C.F.R. part 46.102(l)(2), 21 C.F.R. part 56; 42 U.S.C. Sect. 241(d); 5 U.S.C. Sect. 552a; 44 U.S.C. Sect. 3501 et seq.

§§ The proportions of adolescent ED visits for drug overdose were similar in summer 2022 and 2021. Among males specifically, ED visits for mental health conditions overall, suicide-related behaviors, and drug overdoses were a similar proportion of all ED visits in summer 2022 compared with summer 2021.

¶¶
https://www.cdc.gov/drugoverdose/nonfatal/dashboard/index.html

***
https://www.cdc.gov/nchs/nvss/vsrr/drug-overdose-data.htm

†††
https://www.dea.gov/stories/2021/2021-07/2021-07-23/counterfeit-drugs-social-media

§§§
https://www.cdc.gov/injury/priority/aces.html

¶¶¶
https://www.cdc.gov/suicide/pdf/preventionresource.pdf

****
https://www.cdc.gov/drugoverdose/pdf/pubs/2018-evidence-based-strategies.pdf

††††
https://www.cdc.gov/violenceprevention/pdf/preventingACES.pdf

§§§§ Essentials for Parenting is a free online resource for parents based on the best available evidence on parenting. There are two versions: one for parents of toddlers and preschoolers (https://www.cdc.gov/parents/essentials/index.html) and one for parents of adolescents (https://www.cdc.gov/parents/essentials/teens/index.html). Other resources are also available, such as those from The National Academies of Sciences, Engineering, and Medicine (https://nap.nationalacademies.org/resource/other/dbasse/wellbeing-tools/interactive/).

¶¶¶¶ Examples include those from CDC (https://www.cdc.gov/howrightnow/) and the Substance Abuse and Mental Health Services Administration (https://www.samhsa.gov/talk-they-hear-you).

*****
https://www.samhsa.gov/find-help/988

†††††
https://telehealth.hhs.gov/providers/policy-changes-during-the-covid-19-public-health-emergency/

§§§§§ The U.S. Preventative Services Task Force recommends screening for major depressive disorder (persons aged 12–18 years; https://www.uspreventiveservicestaskforce.org/uspstf/recommendation/screening-depression-suicide-risk-children-adolescents#citation32) and anxiety (persons aged 8–18 years; https://www.uspreventiveservicestaskforce.org/uspstf/recommendation/screening-anxiety-children-adolescents).

¶¶¶¶¶ Example resources for clinicians include those related to children’s mental health (https://www.cdc.gov/childrensmentalhealth/documents/access-infographic.html) and opioid prescribing (https://www.cdc.gov/opioids/patients/guideline.html).

******
https://www.cdc.gov/mentalhealth/stress-coping/help-children-cope/index.html

††††††
https://www.cdc.gov/healthyyouth/whatworks/what-works-safe-and-supportive-environments.htm

§§§§§§
https://www.cdc.gov/violenceprevention/childabuseandneglect/essentials/
